# A Case of Chronic Granulomatous Disease with a Necrotic Mass in the Bronchus: A Case Report and a Review of Literature

**DOI:** 10.1155/2012/980695

**Published:** 2012-12-13

**Authors:** Ali Cheraghvandi, Majid Marjani, Saeid Fallah Tafti, Logman Cheraghvandi, Davoud Mansouri

**Affiliations:** ^1^Chronic Respiratory Diseases Research Center, National Research Institute of Tuberculosis and Lung Diseases (NRITLD), Shahid Beheshti University of Medical Sciences, Tehran 19558 41452, Iran; ^2^Clinical Tuberculosis and Epidemiology Research Center, National Research Institute of Tuberculosis and Lung Disease (NRITLD), Masih Daneshvari Hospital, Shahid Beheshti University of Medical Sciences, Tehran 19558 41452, Iran; ^3^Nursing and Respiratory Health Management Research Center, National Research Institute of Tuberculosis and Lung Diseases (NRITLD), Shahid Beheshti University of Medical Sciences, Tehran 19558 41452, Iran; ^4^Lung Transplantation Research Center, National Research Institute of Tuberculosis and Lung Diseases (NRITLD), Shahid Beheshti University of Medical Sciences, Tehran 19558 41452, Iran

## Abstract

Chronic granulomatous disease is a rare phagocytic disorder with recurrent, severe bacterial and fungal infections. We describe an unusual case of chronic granulomatous disease manifesting as an invasive pulmonary aspergillosis with an obstructive necrotic mass at the right middle bronchus. The patient was successfully treated with a bronchoscopic intervention for the removal of the obstructive mass and a medical therapy.

## 1. Introduction

Chronic granulomatous disease (CGD) is a rare primary immunodeficiency state. Affected cases are susceptible to special infections including particular fungal and bacterial disease [1, 2], most of them are diagnosed in early childhood [3]. Pneumonia is the most common infection and the most common organism is *Aspergillus* spp. [4]. CGD is a life-threatening disease so early diagnosis of an infection is very important.

## 2. Case Report

A 19-year-old woman was referred to our hospital with complaints of productive cough and massive hemoptysis. One month earlier she had been admitted to a hospital in the northern province of the country with symptoms of fever, weight loss, and bloody sputum. A computed tomography (CT) scan was performed and showed cavitary lesions with nodules in both lungs. Medical therapy for pulmonary infection consisting clindamycin and ceftriaxone was started, but did not respond well to the treatment after 3 weeks. So bronchoscopy was performed which reported an acute severe bronchitis. Culture of bronchoalveolar lavage specimen was negative after a 7-day inoculation and the transbronchial lung biopsy (TBLB) showed pulmonary angiitis granulomatosis compatible with Wegener's granulomatosis. Also antinuclear antibody test (ANA) was positive (1/640) but anti-dsDNA was negative. She was referred to our hospital for a complementary workup.

The patient was a young housewife originally from north Iran. Her illness started 30 days ago with fever, night sweats, weakness, and weight loss of four Kg, that followed with productive cough and hemoptysis, about 20 cc of blood in her sputum for 3 times. She had a normal delivery 5 months ago with a normal infant. She is a nonsmoker, without any history of recent travel or drug abuse. She had no significant medical history except an episode of pyelonephritis when she was 6 years old, recurrent oral aphthous ulcers, and multiple episodes of common cold in the last two years. She had four healthy, living siblings and her parents were not related. Her aunt had pulmonary tuberculosis twenty years ago. 

On admission she was dyspneic and febrile with oral temperature of 39°C. Her blood pressure was 110/60 mm Hg, heart rate was 120/min, and respiratory rate was 20/min. Physical examination was unremarkable except pectus excavatum and bilateral course crackles along with rhonchi in right hemithorax. Peripheral blood leukocyte count was 16300/*μ*L with 65% neutrophil, 10% lymphocyte, 11% monocyte, and 14% eosinophil (total eosinophil count: 2282/*μ*L); hemoglobin and platelet counts were 8.6 mg/dL and 552 × 10^9^ per liter. The erythrocyte sedimentation rate was 125 mm/h. All results of biochemical tests were normal. Also c-ANCA, p-ANCA, anti-PR3 IgG, and anti-MPO IgG were negative.

A chest X-ray was done and showed cavitary lesions and nodules in both lungs. Direct smear of sputum for acid fast bacilli was negative three times. Ciprofloxacin and meropenem were started but no improvement was seen in her condition.

**Figure 1:**
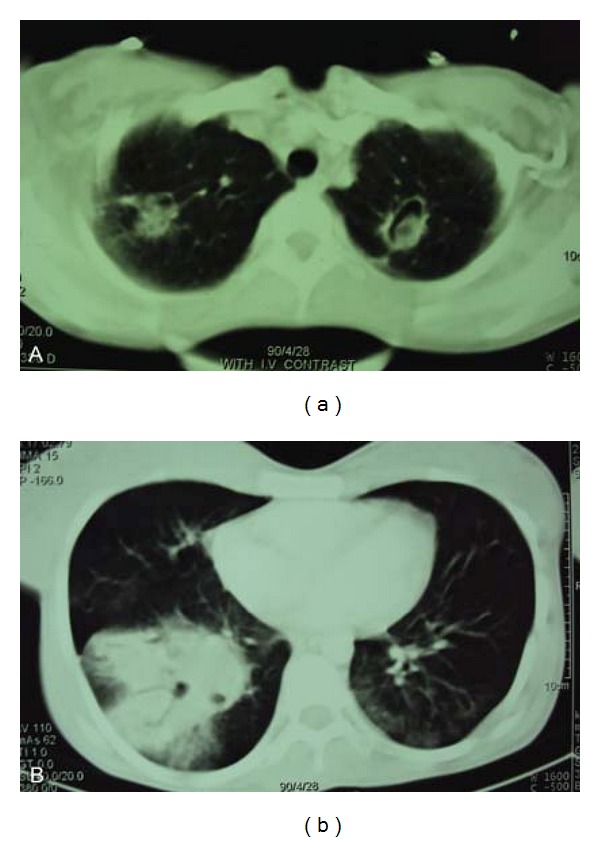
Spiral lung CT scan; (a) biapical cavitary lesions and intracavitary soft tissue projection in the left lung; (b) scattered nodular infiltration with a large mass-like consolidation was seen in the right lower lobe.

The thorax CT scan was performed and showed biapical cavitary lesions and intracavitary soft tissue projection in the left lung. Also scattered nodular infiltration with a large mass-like consolidation was seen in the right lower lobe. Right hilar and subcarinal adenopathy there also reported (Figure 1).

Bronchoscopy was done which showed a necrotic obstructive mass at the right middle bronchus. No bacterial or fungal agents were isolated from sputum and bronchoalveolar lavage (BAL) fluid. Histopathological study showed filamentous septated hyphae with acute angle branching, without any evidence of granuloma or necrotizing vasculitis. Galactomannan assay index of serum was highly positive (10 ng/mL). Voriconazole was added to her treatment.

**Figure 2:**
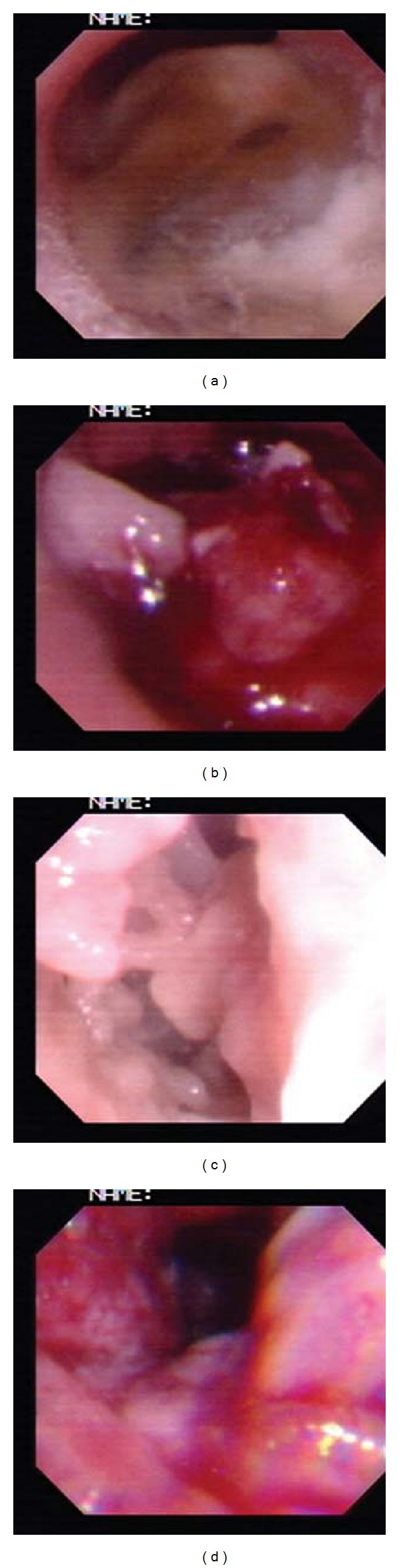
Bronchoscopic view of the right middle bronchus; (a) obstructed bronchus; (b) mass lesion; (c) necrotic material behind mass; (d) after the removal of necrotic material.

*Aspergillus* fumigatus was isolated from the culture of tissue specimen. Bronchoscopy was repeated for the removal of obstructive mass. Distal to it, a lot of necrotic material was discovered (Figure 2). Voriconazole was changed to Itraconazole 200 mg t.i.d after seven days of treatment. Brain CT scan was performed and was normal.

The patient's immune system was assessed. Ranges of the immunoglobulins were normal. The nitroblue-tetrazolium (NBT) test was 0%. Also the dihydrorhodamine 123 (DHR) assay confirmed the diagnosis of chronic granulomatous disease (CGD).

Patient's symptoms improved gradually, fever stopped, and she did not have any episodes of hemoptysis at all. Therapeutic dose of Itraconazole was continued for four months, after that it was prescribed as 100 mg once daily concomitant with Trimethoprim-sulfamethoxazole as prophylaxis up to now. Also at the follow-up visits there was no sign of recurrent infection. Screening of the other members of the family was negative for CGD. 

## 3. Discussion

CGD was for the first time described half a century ago. It is a primary immunodeficiency state of phagocytic cells. CGD now consists of five genetic defects, related to every subunit of the five essential subunits of the phagocyte nicotinamide adenine dinucleotide phosphate (NADPH) oxidase. This enzyme generates reactive oxygen essential for the killing of some bacteria and fungi. Susceptibility to a particular spectrum of infectious agents associated with hyperinflammation and tissue granuloma formation are the characteristics of CGD [1]. 

Overall prevalence of CGD is about 1 in 200000 to 250000 persons. Approximately 70% of them are X-linked variant of disease (gp 91 deficient), and the remainder are autosomal recessive [2].

The majority of patients are diagnosed in early childhood [3], 76% before five years of age but 15% of patients are diagnosed in the second decade of the life [4] and on rare occasions (4%), even later [5, 6].

The X-linked form of disease presents earlier and more severe and rates of infection and death are higher [1]. In contrast, autosomal recessive forms are diagnosed in the older age and have a better outcome [2, 4, 7]. At present, about 50% of CGD cases survive until the third or fourth decades of life [8, 9].

Pneumonia is the most common complication [4], after that skin, lymph nodes, and liver infections are common. Among CGD patients five main groups of pathogens are more frequent: *Aspergillus*, *Staphylococcus aureus*, *Burkholderia*, *Serratia marcescens*, and *Nocardia* [1]. The most common organism is *Aspergillus* spp. [4]. Among them, *Aspergillus nidulans* is a highly likely pathogen, in contrast to infrequency among other immune deficient states [3, 10]. Consequently, the microbiological investigation for infectious agents should be performed and can be highly suggestive of CGD as the underlying condition [3].

One-third of CGD patients present a variety of inflammatory diseases, such as obstructive lesions of gastrointestinal tract and urinary systems. Also inflammatory bowel disease similar to Crohn's disease has been described among them [1, 11].

In this setting, an invasive fungal pneumonia is insidious in onset, although mortality is very high. Patients usually report chronic cough and malaise. Invasive aspergillosis is characterized by impaired ability to damage hyphae, dysregulated inflammation, and local extension to the pleura and the chest wall among one-third of the patients [12, 13]. 

The first element in the care of CGD patients is the early diagnosis of the infection; concerning that the classic signs of infection such as fever and leukocytosis may be absent. An elevated ESR rate may be the only indicator [2]. On the other hand, some specific opportunistic infections including invasive mold diseases and infections by *B. cepacia*, *S. marcescens*, and *Nocardia* species should prompt a diagnostic approach for CGD [2].

 Dihydrorhodamine 1,2,3 (DHR) assay is a flow cytometry technique for the diagnosis of CGD. It is easy and more sensitive than the nitroblue-tetrazolium method (NBT) test [3]. Also in some cases of autosomal recessive and variant X-linked forms of disease, low levels of NADPH oxidase activity may lead to incorrect results with NBT [2]. So DHR is preferred. DHR (dihydrorhodamine 1,2,3) enters phagocytes freely and is oxidized in the cells to rhodamine 1,2,3 by diffusible H_2_O_2_ after phagocytes are stimulated, which is detectible by flow cytometry [1].

During any episode of infection, every attempt should be performed for a microbiological diagnosis. Exclusion of disseminated infection, especially the involvement of central nervous system, is necessary in the cases of pneumonia caused by *Aspergillus* or *Nocardia* spp. that can be performed best by an imaging study [12]. Serum galactomannan (an *Aspergillus* antigen and a diagnostic marker for an invasive disease) appears to be insensitive among CGD patients [2].

All episodes of infection should be treated as soon as possible. Due to slow response, prolonged treatment may be necessary particularly for fungal infections (e.g., for 4–6 months). Antibiotic and antifungal prophylaxis is recommended indefinitely to prevent the recurrence or reactivation of infection. Trimethoprim-sulfamethoxazole and Itraconazole are preferred agents [12]. Cure can be achieved by haematopoietic stem cell transplantation and gene therapy [1].

The above case has some interesting characteristics. She was a 19-year-old woman, without any history of serious infections compatible with the autosomal recessive form of CGD. Typically after the detection of the *Aspergillus* as the cause of infection, CGD was introduced. Although, for the diagnosis of CGD, DHR assay is better, in our setting NBT test is more accessible. She had a history of recurrent aphthous ulcers; this may be related to CGD or not. Aphtus lesions have been reported in gp91 carriers (X-linked form of disease) [14].

A necrotic obstructive mass was observed at right middle bronchus. It may be due to hyper inflammation, a characteristic of *Aspergillus* infection among CGD cases. This mass was removed by interventional bronchoscopy as part of treatment. Although necrotizing aspergillosis of the large airways (necrotizing *Aspergillus* bronchitis) is a known entity [15], to our knowledge, until now, this form of aspergillosis has not been reported among CGD cases in the literature.

## 4. Conclusion

CGD can be a lethal disease. Early diagnosis of the underlying disorder of immune system is very important. We have to consider CGD in any patient with recurrent episodes of severe infections. On the other hand, the isolation of particular opportunistic infections such as *Aspergillus*, *Serratia*, *Nocardia*, and *Burkholderia* should prompt a diagnostic approach for CGD.
